# Empagliflozin and Dapagliflozin Therapies Favorably Alter QRS-T Angle and Cardiac Repolarization Parameters in Patients with Heart Failure

**DOI:** 10.19102/icrm.2024.15044

**Published:** 2024-04-15

**Authors:** Yasin Özen, Mustafa Bilal Özbay, Bede N. Nriagu, İdris Yakut, Yücel Kanal, Elif Hande Özcan Çetin, Ahmet Afsin Oktay

**Affiliations:** 1 Department of Cardiology, Selcuk University, Faculty of Medicine, Konya, Turkey; 2 Department of Internal Medicine, Metropolitan Hospital Center New York Medical College, New York, NY, USA; 3 Department of Cardiology, Medipol Bahçelievler Hospital, Cardiology, Istanbul, Turkey; 4 Department of Cardiology, Cumhuriyet University, Faculty of Medicine, Sivas, Turkey; 5 Department of Cardiology, Ankara City Hospital, Training and Research Hospital, Cardiology, Ankara, Turkey; 6 Department of Medicine, Division of Cardiology, Rush University Medical Center, Chicago, IL, USA

**Keywords:** Cardiac repolarization, frontal-plane QRS-T angle, heart failure, sodium–glucose cotransporter-2 inhibitors

## Abstract

Recent randomized clinical trials demonstrated that treatment with sodium–glucose cotransporter-2 inhibitors (SGLT2is) reduces the risk of cardiac mortality due to sudden cardiac death and progressive pump failure in patients with heart failure (HF). Mechanisms underlying the potential anti-arrhythmic effects of SGLT2is are not well understood. We aimed to examine the effect of SGLT2i treatment on the frontal-plane QRS-T (f[QRS-T]) angle, a novel marker of myocardial repolarization and an independent predictor of adverse cardiac outcomes. The study included 106 patients with HF with reduced ejection fraction (HFrEF) who received an SGLT2i, empagliflozin, or dapagliflozin. All study participants underwent screening 12-lead electrocardiography (ECG) before and ∼90 days after treatment. We compared ECG repolarization parameters before and after treatment. During study enrollment, there were statistically significant decreases in the Tp-e/QT ratio (*P* ≤ .0001), Tp-e/corrected QT ratio (*P* = .0002), Tp-e interval (*P* < .0001), and f(QRS-T) angle (*P* = .04) in response to SGLT2i therapy. In addition, study participants experienced an improvement in functional capacity (2.06 ± 0.6 vs. 1.82 ± 0.6, *P* = .0001) and reduced N-terminal pro-b-type natriuretic peptide values. In this retrospective cohort study, SGLT2i therapy was associated with improved cardiac repolarization parameters in patients with HFrEF. More comprehensive studies are needed to evaluate the impact of SGLT2i on cardiac repolarization and its potential relation to cardiac arrhythmia and sudden cardiac death risk.

## Introduction

Patients with heart failure (HF) with reduced ejection fraction (HFrEF) have a substantially increased risk of sudden cardiac death (SCD) due to ventricular arrhythmias.^1,2^ Sodium–glucose cotransporter-2 inhibitors (SGLT2is) act by inhibiting sodium and glucose reabsorption in the proximal tubules of the kidneys.^3^ Numerous randomized clinical trials (RCTs) have confirmed that these antidiabetic agents have strong cardiorenal benefits independent of their glucose-lowering effects. The EMPagliflozin outcomE tRial in Patients with chrOnic heaRt Failure with Reduced Ejection Fraction (EMPEROR-Reduced)^4^ and Dapagliflozin and Prevention of Adverse outcomes in Heart Failure (DAPA-HF)^5^ studies showed that, compared to placebo, SGLT2i (empagliflozin and dapagliflozin, respectively) therapy was associated with a significant reduction in HF hospitalizations and cardiovascular disease (CVD) mortality, independent of diabetes status. These trial findings led to the establishment of SGLT2is as part of guideline-directed medical therapy (GDMT) for HF.^6,7^

Emerging data have suggested that SGLT2i therapy may reduce the risk of cardiac arrhythmias. For example, a post hoc analysis of the Dapagliflozin Effect on Cardiovascular Events—Thrombolysis in Myocardial Infarction 58 (DECLARE-TIMI 58) trial revealed that dapagliflozin therapy could reduce the risk of atrial fibrillation in high-risk patients with diabetes.^8^ Similarly, data from population-based cohort studies and meta-analyses of RCTs have demonstrated that SGLT2i therapy is associated with a reduced risk of cardiac arrhythmias (ie, atrial fibrillation and ventricular tachycardia) and SCD.^9–13^ However, the potential mechanisms underlying this effect are not yet understood.

Delayed ventricular repolarization is associated with an increased risk of ventricular arrhythmias.^14^ Ventricular repolarization can be determined on standard 12-lead electrocardiography (ECG) using QT dispersion, QT interval, and T-wave measurements.^15^ Recent studies have suggested that the Tp-e interval, which is the interval between the peak and end of the T-wave, can be a marker of total dispersion of repolarization.^16,17^ Furthermore, a prolonged Tp-e interval may predict the risk of ventricular arrhythmias and mortality.^18^ Hence, the Tp-e/QT ratio was suggested to be an independent marker of ventricular repolarization.

The spatial QRS-T angle, defined as the angle difference between the direction of ventricular depolarization (QRS wave) and the direction of ventricular repolarization (T-wave), is a new marker of myocardial repolarization.^19^ Moreover, measuring the spatial QRS-T angle is extremely difficult and necessitates the use of sophisticated computer programs.^20^ On the contrary, the QRS-T angle in the frontal plane is easily measured from the automatic report portion of ECG devices and correlates well with the spatial QRS-T angle in risk estimation.^21^ Therefore, the frontal QRS-T angle has received more attention than the spatial QRS-T angle. The frontal-plane QRS-T (f[QRS-T]) angle is defined as the angle difference between the directions of ventricular depolarization (QRS) and repolarization (T) axes on a 12-lead ECG.^22^ The f(QRS-T) angle is an indicator of ventricular repolarization heterogeneity, and widened f(QRS-T) has been shown to predict an increased risk of ventricular arrhythmias and CVD mortality.^19^ The prognostic value of this readily available parameter has been shown in different populations.^19,23^ In a cohort of 467 patients with acute myocardial infarction and left ventricular systolic dysfunction, a widened f(QRS-T) angle (>90°) was a significant discriminator of long-term mortality risk.^24^

In light of recent publications demonstrating the potential anti-arrhythmic benefits of SGLT2is,^13^ we hypothesized that SGLT2i therapy might affect cardiac repolarization parameters. Therefore, we aimed to investigate the potential relationship between SGLT2i treatment and cardiac repolarization parameters in patients with HFrEF.

## Materials and methods

### Population and sample

We retrospectively analyzed a cohort of 144 consecutive patients with HFrEF (EF ≤ 35%) who were cared for in the outpatient setting at an academic tertiary health care center between September 2021 and October 2022. We included patients with a New York Heart Association functional class of 1–3 and a previous diagnosis of HFrEF who were on optimal GDMT and started on an SGLT2i (10 mg orally daily of either empagliflozin or dapagliflozin) as part of HF management. Patients with basal creatine levels of ≥1.5 mg/dL were not included in the study. In addition, we later excluded 37 patients who had been initially enrolled, including 12 who developed acute kidney injury (defined by an increase in serum creatinine of ≥0.3 mg/dL within 48 h or ≥50% within 7 days or a urine output of <0.5 mL/kg/h for >6 h),^25^ 7 with SCD, and 18 who were not on SGLT2i maintenance therapy during follow-up due to medication non-compliance or financial reasons. Additional exclusion criteria included ECG evidence of bundle branch block, atrioventricular conduction abnormalities, U-waves, and difficulty analyzing QT and Tp-e intervals. As a result, we finally enrolled a total 106 patients in our analysis.

All patients’ demographic, ECG, and echocardiographic data were collected from clinical follow-up visits, patient files, and the electronic database. All patients underwent 12-lead ECG recordings and average N-terminal pro-b-type natriuretic peptide (NT-proBNP) testing at the initial and ∼90-day clinical follow-up visits. Hypertension was defined as a blood pressure of ≥140/90 mmHg during at least three different measurements or a history of antihypertensive medication use. Diabetes mellitus was defined by a fasting blood glucose level of ≥126 mg/dL, glycated hemoglobulin (HbA1c) value of >6.4, or a history of antidiabetic drug use. Hyperlipidemia was defined by a total cholesterol level of ≥200 mg/dL or a history of statin therapy. The echocardiographic assessment was performed using an iE33 xMATRIX Cardiovascular Ultrasound System (Koninklijke Philips N.V., Amsterdam, the Netherlands) with a 3.5-MHz transducer. The modified Simpson method was used to calculate EF.

### Electrocardiography

**Figure 1:**
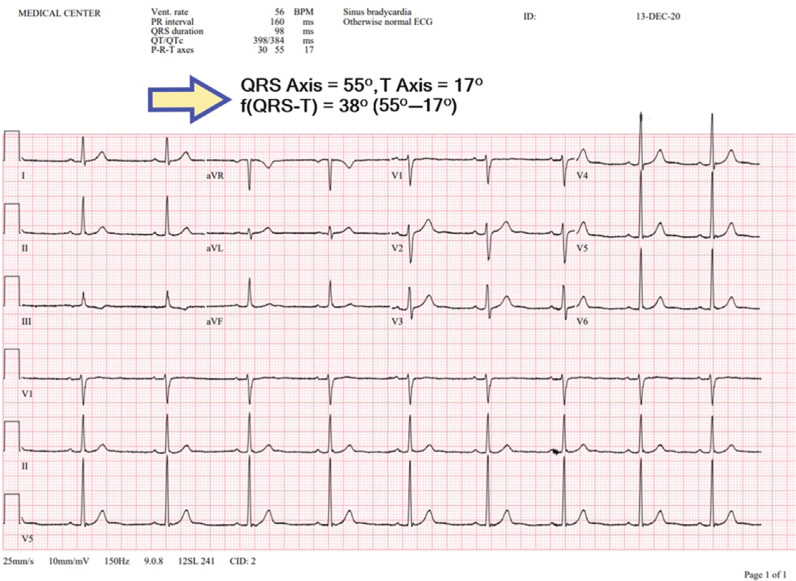
Example of QRS-T angle calculation.

A 12-lead ECG was recorded in the supine position at a paper speed of 50 mm/s (Nihon Kohden, Tokyo, Japan). All ECG records were scanned into electronic format using Adobe Photoshop (Adobe Inc., San Jose, CA, USA) and magnified by 400% to reduce errors. QT and Tp-e intervals on ECGs were measured by two cardiologists blinded to the patients’ data. QT interval was measured from the beginning of the QRS complex to the end of the T-wave and then corrected for heart rate using Bazett’s formula: cQT = QT√(R–R interval). The Tp-e interval in the precordial leads (V_1_–V_6_) was measured as described previously.^15,26^ The f(QRS-T) angle was calculated as the absolute difference between frontal QRS and T-wave axes automatically derived from the ECG machine. If the f(QRS-T) angle was >180°, the value was subtracted from 360^19,22,23,27^ **(Figure 1)**. We preferred machine-derived QRS and T-wave axes to calculate the f(QRS-T) angle to rule out measurement subjectivity.

### Statistical analysis

We calculated the mean ± standard deviation values and percentages for continuous and categorical variables, respectively (ie, demographic variables, co-morbidities, and other clinical parameters). We recorded the NT-proBNP value; the New York Heart Association functional class; and computed electrocardiographic indices including QT interval, QTc interval, Tp-e interval, Tp-e/QT ratio, Tp-e/QTc ratio, and f(QRS/T) (°) both pre- and post-SGLT2i treatment. A paired-samples *t* test was used to compare the mean NT-proBNP value, functional class, and ECG indices pre- and post-SGLT2i treatment. *P* < .05 was considered to indicate statistical significance. Analyses were conducted using PROC FREQ and PROC TTEST in SAS version 9.4 (SAS Institute, Inc., Cary, NC, USA). The study was carried out per the ethical principles specified in the Declaration of Helsinki, Good Clinical Practice, and International Conference on Harmonization guidelines. Before the study, written informed consent was obtained from all participants, and the local ethics committee approved the study protocols.

## Results

The demographic and clinical characteristics of the study group are shown in **Table 1**. The mean age was 56.5 ± 9.4 years, and 35.9% of patients were female. The mean body mass index was 27.6 ± 2.3 kg/m^2^. The mean EF was 28.98% ± 3.98%. During study enrollment, patients’ functional capacity improved (2.06 ± 0.6 vs. 1.82 ± 0.6, *P* = .0001) and their NT-proBNP values decreased (2736.7 ± 2101.0 vs. 2439.1 ± 1673.4 pg/mL, *P* = .0029) **(Table 2)**. There was no statistically significant difference between pre- and post-therapy QT (*P* = .194) and corrected QT intervals (*P* = .459). However, after ∼90 days of SGLT2i therapy, there was a statistically significant decrease in the Tp-e/QT ratio (*P* <. 0001), Tp-e/corrected QT ratio (*P* = .0002), Tp-e interval (*P* < .0001), and QRS-T angle (*P* = .04), respectively.

**Table 1:** Demographic and Echocardiographic Features of the Study Group

Parameters	(N = 106)
Age, years	56.45 ± 9.40
Female, n (%)	38 (35.9%)
BMI, kg/m^2^	27.6 ± 2.3
Ischemic etiology, n (%)	80 (75.5%)
Hypertension, n (%)	40 (37.7%)
Diabetes mellitus, n (%)	38 (35.9%)
Dyslipidemia, n (%)	32 (30.2%)
Smoking, n (%)	38 (35.9%)
Ejection fraction, %	28.98 ± 3.98
NYHA functional class	2.06 ± 0.60
Beta-blocker therapy, n (%)	96 (90.6%)
ACEi or ARB therapy	98 (92.5%)
Mineralocorticoid receptor antagonists, n (%)	84 (79.3%)
Ivabradine, n (%)	28 (26.4%)
Thiazide diuretics, n (%)	34 (32.1%)
Total furosemide dosage, mg	31.7 ± 25.7

*Abbreviations:* ACEi, angiotensin-converting enzyme inhibitor; ARB, angiotensin receptor blocker; BMI, body mass index; NYHA, New York Heart Association; SD, standard deviation. Data are given as mean ± standard deviation, n, or median (interquartile range).

**Table 2:** Comparison of Functional Class, NT-proBNP, and ECG Parameters Before and After SGLT2i Therapy

Parameters	Before therapy	∼90-day follow-up	*P* value
NYHA functional class	2.06 ± 0.6	1.82 ± 0.6	.0001*
NT-proBNP, pg/mL	2736.7 ± 2101.0	2439.1 ± 1673.4	.0029*
QT interval, ms	353.2 ± 28.0	357.3 ± 28.4	.194
QTc interval, ms	384.7 ± 35.4	381.2 ± 34.5	.459
Tp-e interval, ms	85.9 ± 11.9	79.9 ± 12.1	<.0001*
Tp-e/QT ratio	0.25 ± 0.04	0.22 ± 0.04	<.0001*
Tp-e/QTc ratio	0.23 ± 0.04	0.21 ± 0.04	.0002*
f(QRS/T) (°)	72.2 ± 27.6	63.5 ± 30.9	.04*

*Abbreviations:* f(QRS/T), frontal-plane QRS-T angle; NYHA, New York Heart Association; NT-proBNP, N-terminal pro-b-type natriuretic peptide; QTc, corrected QT interval. Data are given as mean ± standard deviation. *Statistically significant.

## Discussion

The main findings of the present study are as follows: HFrEF patients who were treated with an SGLT2i experienced (1) improvement of repolarization dispersion–assessed Tp-e interval, Tp-e/QT ratio, Tp-e/QTc ratio, and f(QRS/T) (°) and (2) no change in QT or corrected QT intervals during the ∼90-day follow-up period. As expected, our study cohort also experienced an improvement in functional class and a reduction in NT-proBNP values during the study period. As far as we can tell, based on our extensive literature search, ours is the first cohort study specifically evaluating the relationship between SGLT2i therapy and electrocardiographic repolarization parameters in patients with HFrEF.

HF is a major cause of morbidity and mortality. Although some data suggest that the incidence of HF is mostly stable or declining, the burden of mortality and number of hospitalizations have not been reduced, despite advances in managing HF.^28^ Furthermore, despite effective disease-modifying GDMT options, patients with HFrEF are at increased risk of cardiac arrhythmias and SCD.^4,5,29,30^ Moreover, we still need well-established tools beyond conventional parameters (EF or QT interval) to predict arrhythmia risk.^31^

The f(QRS-T) angle is a new marker used to measure the heterogeneity of ventricular repolarization.^22^ As the axes of myocardial depolarization and repolarization are similar, the f(QRS-T) angle tends to be narrow (<45°). Therefore, a wide f(QRS-T) angle indicates the abnormality between ventricular depolarization and repolarization phases.^32^ The clinical significance of the f(QRS-T) angle was demonstrated in several cohort studies. The QRS-T angle has been reported to have more additive value in identifying problems than other conventional parameters for myocardial repolarization on ECG.^33,34^

Previous studies suggested that f(QRS-T) has a value similar to spatial QRS-T angle in predicting cardiovascular mortality.^21^ A wide f(QRS-T) angle (>90°) was found to be an independent predictor of long-term mortality in a cohort of patients with acute myocardial infarction and reduced EF.^24^ Slow coronary flow was associated with wider f(QRS-T) and prolonged Tp-e interval, Tp-e/QT, and Tpe/QTc ratios compared to those in the general population in a retrospective cohort study.^35^ Okutucu et al. observed that switching from ramipril to sacubitril/valsartan therapy improved some repolarization by decreasing QTc, Tp-e, and Tp-e/QTc in patients with HFrEF.^36^

Over the past decade, the cardioprotective effects of SGLT2is have been increasingly recognized thanks to consistent results from numerous cardiovascular outcome trials. It has been hypothesized that SGLT2i therapy may partly reduce the risk of cardiovascular mortality by exerting a favorable effect on cardiac arrhythmia risk.^9^ In a recent secondary analysis of the DECLARE-TIMI 58 study, dapagliflozin reduced the risk of atrial flutter/fibrillation by 19% compared to placebo.^8^ On the contrary, in the Empagliflozin Cardiovascular Outcome Event Trial in patients with Type 2 Diabetes Mellitus (EMPA-REG OUTCOME), the incidence of atrial fibrillation was higher in the empagliflozin arm (2.3%) than in the placebo arm (1.6%), but no significant differences were observed between groups.^37^ The most common side effects due to SGLT2is are reported to be volume depletion and urinary and genital system infections. Less frequently, hypoglycemia, diabetic ketoacidosis, amputations of the lower extremities, bone fractures, and Fournier gangrene may occur. However, SGLT2is were well tolerated both EMPA-REG and DECLARE TIMI-58.^38^

The impact of SGLT2i therapy on other cardiac arrhythmias, such as ventricular tachycardia (VT), has not been studied extensively. A meta-analysis including 22 RCTs conducted by Li et al.^9^ found that SGLT2i therapy may be associated with a lower risk of atrial flutter/fibrillation and ventricular tachycardia compared to placebo. In this meta-analysis, the majority of trials included patients with diabetes and chronic kidney disease, and two trials enrolled patients with HF. A population-based propensity score–matched cohort study from Taiwan, including 79,150 diabetic patients treated with SGLT2is compared with matched diabetic patients not taking SGLT2is, reported a 45% reduction in the adjusted risk of all-cause death and a 17% reduction in the risk of new-onset arrhythmias with the use of SGLT2i therapy.^11^

Mechanisms underlying the anti-arrhythmic effects of SGLT2is remain poorly understood. However, several mechanisms have been proposed to play a role in the reduction of arrhythmia risk in response to SGLT2i therapy, such as hemodynamic effects that reduce preload and afterload by decreasing plasma volume and blood pressure,^39–41^ the inhibition of sodium–hydrogen exchange in myocardial cells associated with reduced myocardial hypertrophy, fibrosis, adverse remodeling, improved systolic dysfunction, and sympathetic nervous system inhibition.^39,42^ In addition to these proposed mechanisms, our results may indicate a potential role of SGLT2i therapy in improving cardiac repolarization. However, more investigation is needed to understand whether these results would translate into clinical outcomes, such as a reduced risk of arrhythmia or mortality.

### Limitations of the study

Our study has several limitations. First, our study had a retrospective design with a relatively small sample size and short duration of follow-up, and it did not have a control arm. Therefore, we cannot confirm that the observed associations are solely due to SGLT2i therapy rather than chance. In addition, our patients were recruited from a single center, and our findings would need further validation in different populations. Another limitation of our study was the underrepresentation of female patients, which may also limit the generalizability of our findings. Finally, as mentioned above, our study used an ECG surrogate for arrhythmia risk, and the clinical significance of our findings needs to be tested in future studies evaluating clinical outcomes.

## Conclusion

In this retrospective cohort study, we observed a statistically significant improvement in repolarization parameters with the initiation and maintenance of SGLT2i therapy in patients with HFrEF. Our results may indicate potential anti-arrhythmic effects of SGLT2i therapy through improved cardiac repolarization in patients with HF. Because of the limitations of our study, our findings could be considered hypothesis-generating, and our results will need further validation in larger and more comprehensive studies.

## References

[1] Koplan BA, Stevenson WG (2009). Ventricular tachycardia and sudden cardiac death. Mayo Clinic Proc.

[2] Singh SN, Carson PE, Fisher SG (1997). Nonsustained ventricular tachycardia in severe heart failure. Circulation.

[3] Zelniker TA, Braunwald E (2018). Cardiac and renal effects of sodium-glucose co-transporter 2 inhibitors in diabetes: JACC state-of-the-art review. J Am Coll Cardiol.

[4] Packer M, Anker SD, Butler J (2020). Cardiovascular and renal outcomes with empagliflozin in heart failure. N Engl J Med.

[5] McMurray JJV, Solomon SD, Inzucchi SE (2019). Dapagliflozin in patients with heart failure and reduced ejection fraction. N Engl J Med.

[6] McDonagh TA, Metra M, Adamo M (2022). 2021 ESC Guidelines for the diagnosis and treatment of acute and chronic heart failure: developed by the Task Force for the diagnosis and treatment of acute and chronic heart failure of the European Society of Cardiology (ESC). With the special contribution of the Heart Failure Association (HFA) of the ESC. Eur J Heart Fail.

[7] Heidenreich PA, Bozkurt B, Aguilar D (2022). 2022 AHA/ACC/HFSA Guideline for the management of heart failure: a report of the American College of Cardiology/American Heart Association Joint Committee on Clinical Practice Guidelines. J Am Coll Cardiol.

[8] Zelniker TA, Bonaca MP, Furtado RHM (2020). Effect of dapagliflozin on atrial fibrillation in patients with type 2 diabetes mellitus: insights from the DECLARE-TIMI 58 trial. Circulation.

[9] Li HL, Lip GYH, Feng Q (2021). Sodium-glucose cotransporter 2 inhibitors (SGLT2i) and cardiac arrhythmias: a systematic review and meta-analysis. Cardiovasc Diabetol.

[10] Bonora BM, Raschi E, Avogaro A, Fadini GP (2021). SGLT-2 inhibitors and atrial fibrillation in the Food and Drug Administration adverse event reporting system. Cardiovasc Diabetol.

[11] Chen HY, Huang JY, Siao WZ, Jong GP (2020). The association between SGLT2 inhibitors and new-onset arrhythmias: a nationwide population-based longitudinal cohort study. Cardiovasc Diabetol.

[12] Persson F, Nyström T, Jørgensen ME (2018). Dapagliflozin is associated with lower risk of cardiovascular events and all-cause mortality in people with type 2 diabetes (CVD-REAL Nordic) when compared with dipeptidyl peptidase-4 inhibitor therapy: a multinational observational study. Diabetes Obes Metab.

[13] Fernandes GC, Fernandes A, Cardoso R (2021). Association of SGLT2 inhibitors with arrhythmias and sudden cardiac death in patients with type 2 diabetes or heart failure: a meta-analysis of 34 randomized controlled trials. Heart Rhythm.

[14] Vaughan Williams EM (1985). Delayed ventricular repolarization as an anti-arrhythmic principle. Eur Heart J.

[15] Castro Hevia J, Antzelevitch C, Tornés Bárzaga F (2006). Tpeak-Tend and Tpeak-Tend dispersion as risk factors for ventricular tachycardia/ventricular fibrillation in patients with the Brugada syndrome. J Am Coll Cardiol.

[16] Antzelevitch C, Sicouri S, Di Diego JM (2007). Does Tpeak-Tend provide an index of transmural dispersion of repolarization?. Heart Rhythm.

[17] Floré V, Willems R (2012). T-wave alternans and beat-to-beat variability of repolarization: pathophysiological backgrounds and clinical relevance. Acta Cardiol.

[18] Panikkath R, Reinier K, Uy-Evanado A (2011). Prolonged Tpeak-to-tend interval on the resting ECG is associated with increased risk of sudden cardiac death. Circ Arrhythm Electrophysiol.

[19] Oehler A, Feldman T, Henrikson CA, Tereshchenko LG (2014). QRS-T angle: a review. Ann Noninvasive Electrocardiol.

[20] Okin PM (2006). Electrocardiography in women: taking the initiative. Circulation.

[21] Zhang ZM, Prineas RJ, Case D, Soliman EZ, Rautaharju PM (2007). Comparison of the prognostic significance of the electrocardiographic QRS/T angles in predicting incident coronary heart disease and total mortality (from the atherosclerosis risk in communities study). Am J Cardiol.

[22] Macfarlane PW (2012). The frontal plane QRS-T angle. Europace.

[23] Aro AL, Huikuri HV, Tikkanen JT (2012). QRS-T angle as a predictor of sudden cardiac death in a middle-aged general population. Europace.

[24] Raposeiras-Roubín S, Virgós-Lamela A, Bouzas-Cruz N (2014). Usefulness of the QRS-T angle to improve long-term risk stratification of patients with acute myocardial infarction and depressed left ventricular ejection fraction. Am J Cardiol.

[25] Khwaja A (2012). KDIGO clinical practice guidelines for acute kidney injury. Nephron Clin Pract.

[26] Ibisoglu E, Boyraz B (2021). Comparison of ventricular repolarization parameters of Covid-19 patients diagnosed with chest CT and RT-PCR. Acta Cardiol.

[27] Tanriverdi Z, Besli F, Gungoren F (2018). Frontal QRS-T angle as a marker of left ventricular hypertrophy in patients with essential hypertension. Dokuz Eylül Üniversitesi Tıp Fakültesi Dergisi.

[28] Roger VL (2021). Epidemiology of heart failure: a contemporary perspective. Circ Res.

[29] Armstrong PW, Pieske B, Anstrom KJ (2020). Vericiguat in patients with heart failure and reduced ejection fraction. N Engl J Med.

[30] McMurray JJ, Packer M, Desai AS (2014). Angiotensin-neprilysin inhibition versus enalapril in heart failure. N Engl J Med.

[31] Braschi A, Frasheri A, Lombardo RM (2021). Association between Tpeak-Tend/QT and major adverse cardiovascular events in patients with Takotsubo syndrome. Acta Cardiol.

[32] Gungor M, Celik M, Yalcinkaya E (2017). The value of frontal planar QRS-T angle in patients without angiographically apparent atherosclerosis. Med Princ Pract.

[33] Dilaveris P, Antoniou CK, Gatzoulis K, Tousoulis D (2017). T wave axis deviation and QRS-T angle - controversial indicators of incident coronary heart events. J Electrocardiol.

[34] Zhang ZM, Rautaharju PM, Prineas RJ, Tereshchenko L, Soliman EZ (2017). Electrocardiographic QRS-T angle and the risk of incident silent myocardial infarction in the Atherosclerosis Risk in Communities study. J Electrocardiol.

[35] Kuyumcu MS, Özbay MB, Özen Y, Yayla Ç (2020). Evaluation of frontal plane QRS-T angle in patients with slow coronary flow. Scand Cardiovasc J.

[36] Okutucu S, Sabanoglu C, Yetis Sayin B, Aksoy H, Bursa N, Oto A (2020). Switching from ramipril to sacubitril/valsartan favorably alters electrocardiographic indices of ventricular repolarization in heart failure with reduced ejection fraction. Acta Cardiol.

[37] Böhm M, Slawik J, Brueckmann M (2020). Efficacy of empagliflozin on heart failure and renal outcomes in patients with atrial fibrillation: data from the EMPA-REG OUTCOME trial. Eur J Heart Fail.

[38] Vardeny O, Vaduganathan M (2019). Practical guide to prescribing sodium-glucose cotransporter 2 inhibitors for cardiologists. JACC Heart Fail.

[39] Packer M, Anker SD, Butler J, Filippatos G, Zannad F (2017). Effects of sodium-glucose cotransporter 2 inhibitors for the treatment of patients with heart failure: proposal of a novel mechanism of action. JAMA Cardiol.

[40] Verma S, McMurray JJV (2018). SGLT2 inhibitors and mechanisms of cardiovascular benefit: a state-of-the-art review. Diabetologia.

[41] Cherney DZ, Odutayo A, Aronson R, Ezekowitz J, Parker JD (2019). Sodium glucose cotransporter-2 inhibition and cardiorenal protection: JACC review topic of the week. J Am Coll Cardiol.

[42] Matthews VB, Elliot RH, Rudnicka C, Hricova J, Herat L, Schlaich MP (2017). Role of the sympathetic nervous system in regulation of the sodium glucose cotransporter 2. J Hypertens.

